# The dimmer switch in epigenetics: How DNA methylation encodes gene activity over time

**DOI:** 10.1016/j.xgen.2025.101070

**Published:** 2025-11-12

**Authors:** Aleksandra Pękowska

**Affiliations:** 1 Dioscuri Centre for Chromatin Biology and Epigenomics, Nencki Institute of Experimental Biology, Polish Academy of Sciences, 3 Pasteur Street, 02-093 Warsaw, Poland

## Abstract

Can individual cells retain a memory of past gene expression levels even after the stimulus has faded? In this issue of *Cell Genomics*, Domitilla Del Vecchio and colleagues describe an analog epigenetic memory system in which DNA methylation acts as a signal that locks in gene expression levels over time and cell divisions.^1^

## Main text

Understanding how cells maintain gene expression state through divisions and over time is a fundamental challenge in molecular biology. Epigenetics refers to the preservation of a gene’s transcriptional program without permanent changes to the DNA sequence. DNA methylation and histone modifications play a vital role in this phenomenon.

**Figure 1:**
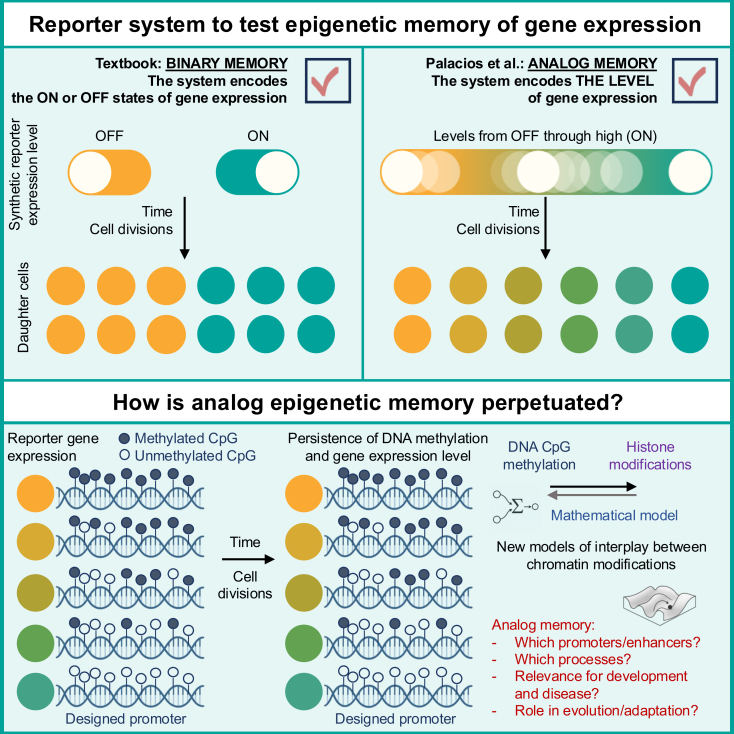
Analog memory of gene expression Epigenetic mechanisms can fix genes in “on” or “off” states, creating binary memory of gene expression (top left). Whether cells can also encode intermediate levels of activity (analog memory) was unclear (top right, colors indicate expression levels). Palacios et al. built a reporter system in which transient recruitment of a DNA methyltransferase to the promoter produced a graded range of stable expression states, from partial repression to complete silencing (bottom). These patterns persisted after enzyme removal, and the level of silencing correlated with the fraction of methylated CpGs. The authors propose a computational model to explore the dynamic interdependencies between DNA methylation and histone modifications in this process, opening new avenues for understanding the implications of analog epigenetic memory in health, disease, development, and evolution.

Early studies revealed that DNA methylation blocks expression,^2^^,^^3^ while demethylation reactivates silent loci, including the inactive X chromosome.^4^^,^^5^ Furthermore, histone-modifying enzymes act as core regulators of transcription and nuclear organization.^6^^,^^7^ Together, these insights advanced a model in which chromatin modifications contribute to preserving ON/OFF (“digital” or “bistable”) expression states over time. But can the chromatin landscape at a locus stably encode not just whether a gene is on or off but also the actual level of its expression? In other words, can epigenetic memory be analog (Figure 1)?

Bulk assays suggested that histone-mark levels are predictive of gene expression^8^ and chromatin states are associated with innate immune “training,”^9^ indicating the possibility of durable graded tuning of future gene expression. Yet, to prove analog epigenetic memory, one would need to provide single-cell, single-locus measurements showing stable intermediate chromatin mark levels that predict proportionate transcription in the same cells and across time. Such data were not available.

In this issue, Palacios and colleagues devise an elegant system to address this question. They assemble a single-copy genomic reporter to test the long-term impact of chromatin modifications on gene expression. Their tool uses the mammalian EF1A promoter, engineered with binding sites for PhlF, rTetR, and guide RNAs, to control expression of a blue fluorescent protein (EBFP2) gene. The construct is inserted between two chromatin insulators, providing a controlled genomic environment.

In this experimental framework, EBFP2 remained active in the steady state. Hence, the authors first introduced DNA methylation at the locus to silence it by transfecting the reporter cells with expression plasmids encoding DNMT3A-dCas9 or rTetR-DNMT3A, along with yellow fluorescent protein. Separating the cells according to distinct levels of yellow signal, reflecting the levels of DNA methyltransferase, followed by the recording of EBFP2 expression in single cells over time, revealed gene silencing. Notably, once established, the cells maintained a constant level of EBFP2 expression over 5 months of tracking, regardless of the long-extinguished presence of the epi-modifier. Thus, DNA methylation can stably encode the level of gene expression.

To further support their findings, the authors track cells with varying EBFP2 expression and monitor DNA (CpG) methylation over time, finding a steady fraction of modified CpGs in each clonal population. To link epigenetic memory to DNA methylation mechanistically, they either remove DNA methylation (using chemical inhibition) or introduce hydroxymethylation at chosen CpGs with dCas9-TET1, leading to local demethylation. Global inhibition fully derepresses EBFP2, while partial demethylation leads to limited reactivation. In contrast, modulating chromatin landscape with dCas9-KRAB, which leads to the deposition of H3K9me3 but does not induce CpG methylation, was not sufficient to encode stable silencing. Therefore, the degree of CpG DNA methylation underlies analog memory of gene expression (Figure 1).

The authors integrated their experimental data with a mathematical model that encodes interactions, turnover rates, and dependencies. This model predicted perturbation outcomes and suggested new relationships among chromatin marks. While these predictions remain to be validated, the framework gives a mechanistic scaffold for exploring analog epigenetic memory. The authors argue that DNA methylation is less subject to strong positive feedback loops compared to histone modification systems. The absence of strong feedback, together with slower turnover kinetics, allows the DNA methylation grade to persist stably, enabling analog memory. Because their model is fitted to data and captures mechanistic dependencies, the authors suggest it can be used to predict outcomes of untested perturbations and to infer plausible relationships between chromatin marks.

Altogether, the authors demonstrate that gene expression can be “memorized” not just in a binary on/off fashion but over a *graded* range of levels. By establishing the plausibility of analog epigenetic memory, this work changes how we think about the temporal control of gene expression.

From a systems point of view, a particular cell fate can be encapsulated as a high-dimensional pattern of gene activity across multiple loci.^10^ It remains to be seen how precisely analog epigenetic memory contributes to the establishment and perpetuation of the network of gene expression in the developing embryo and how it relates to disease states.

Additional mechanistic insights into how CpG content, density, distribution, and sequence context determine a locus’s capacity to store the information on quantitative expression will be instrumental in understanding the nature of this process. Likewise, addressing whether there are other chromatin modifications or transcription factor-binding motifs implicated in storing the information of the level of gene expression will be needed to put analog memory of gene expression in the broader context of transcriptional control. Finally, elucidating how local chromatin environment modulates the capacity to store the information on the level of gene expression, and which biological programs, fate decisions, and disease states depend on analog epigenetic memory of gene activity, will help build refined models of the stable control of gene activity (Figure 1). Some species have little DNA methylation or CpG-sparse genomes: can they also encode graded, time-stable expression epigenetically? Addressing these questions will open new frontiers in epigenetics.

## Acknowledgments

Work in the Pękowska lab is funded by the Dioscuri Grant (Dioscuri is a program initiated by the Max Planck Society [MPG], jointly managed with the National Science Centre in Poland [NCN] and mutually funded by the Polish Ministry of Education and Science and the German Federal Ministry of Education and Research [BMBF] UMO-2018/01/H/NZ4/00001), by the EMBO Installation Grant, and by OPUS17 (UMO-2019/33/B/NZ2/02437), OPUS22 (UMO-2021/43/B/NZ2/02934), and Sonata Bis 11 (UMO-2021/42/E/NZ2/00392) grants from the National Science Centre in Poland and chrom_rare consortium grant (Maria Skłodowska Curie Actions, GA n°101073334-Chrom_rare).

## Declaration of interests

The author declares no competing interests.

## References

[1] Palacios S., Bruno S., Weiss R., Salibi E., Goodchild-Michelman I., Kane A., Ilia K., Del Vecchio D. (2025). Analog epigenetic memory revealed by targeted chromatin editing. Cell Genom..

[2] Stein R., Razin A., Cedar H. (1982). In vitro methylation of the hamster adenine phosphoribosyltransferase gene inhibits its expression in mouse L cells. Proc. Natl. Acad. Sci. USA.

[3] Vardimon L., Kressmann A., Cedar H., Maechler M., Doerfler W. (1982). Expression of a cloned adenovirus gene is inhibited by in vitro methylation. Proc. Natl. Acad. Sci. USA.

[4] Jones P.A., Taylor S.M., Mohandas T., Shapiro L.J. (1982). Cell cycle-specific reactivation of an inactive X-chromosome locus by 5-azadeoxycytidine. Proc. Natl. Acad. Sci. USA.

[5] Mohandas T., Sparkes R.S., Shapiro L.J. (1981). Reactivation of an inactive human X chromosome: Evidence for X inactivation by DNA methylation. Science (1979).

[6] Strahl B.D., Allis C.D. (2000). The language of covalent histone modifications. Nature.

[7] Jenuwein T., Allis C.D. (2001). Translating the Histone Code. Science (1979).

[8] Karlić R., Chung H.-R., Lasserre J., Vlahovicek K., Vingron M. (2010). Histone modification levels are predictive for gene expression. Proc. Natl. Acad. Sci. USA.

[9] Saeed S., Quintin J., Kerstens H.H.D., Rao N.A., Aghajanirefah A., Matarese F., Cheng S.C., Ratter J., Berentsen K., Van Der Ent M.A. (2014). Epigenetic programming of monocyte-to-macrophage differentiation and trained innate immunity. Science (1979).

[10] Huang S., Kriete A., Eils R. (2006). Computational Systems Biology.

